# The Renaissance of the Profession

**Published:** 1916-08

**Authors:** 


**A Monthly Review of Medicine and Surgery**

EDITOR AND PUBLISHER, DR. A. L. BENEDICT, 228 Summer St., corner of Elmwood Ave., Buffalo, (Address for all communications. Please make personal and telephone calls before 1 P. M.)

**Third, in age, on the western continent. Independent, but supports professional organization. News limited mainly to Western N. Y. and Penn.**

**Subscription $2.00 a year; $3.00 for two years in advance. Changes and errors in address or failure or duplication of delivery, should be reported immediately.**

Yearly Volume 72 AUGUST, 1916 Number 1

### The Renaissance of the Profession.

Many excellent persons, with no desire to be pessimistic or disagreeable, find fault much more frequently than they bestow commendation. They are so thoroughly impressed with the importance of maintaining high standards that they take as a matter of course, whatever is good and laudable and protest vehemently against any departure from the ideal. While believing that the best that is humanly speaking attainable, is the normal standard, it does no harm, occasionally, to praise the man whose work is good nor even, within limits to commend ourselves.

In speaking of the Renaissance of the Profession, we have no intention of enumerating the many additions to our scientific knowledge, acquired within the memory of men still living nor of again alluding to the higher standards of general and technical education now inforced. Indeed, during the last few years, we have had occasion to search out early cases of a certain rare condition and, in the search, have been led to read several medical works of the eighteenth and seventeenth century, even reaching into the sixteenth and have lost much of the natural conceit of modern superiority. Not only does it appear that we really do not know a great deal more than our predecessors three centuries ago, but one is impressed with their greater scholarship. In the main, whatever superiority we possess, rests on the availability of various absolute facts, due to co-operation, better knowledge of one another's work., compilation of statistics, and to technical scientific experimentation and research based on essentially extra-medical studies. In reading the old authors, we find an immense amount of erudition that is now useless or false and certain misconceptions now corrected. But, the fair-minded modern can readily imagine that our fathers in medicine would not have made these mistakes, if they had had at hand the cold facts furnished us from other sciences and if they could have compared notes across international lines as easily and quickly as can be done at present. To allude to one detail, it seems that if the medical profession of the twentieth century, had so general a familiarity with the lymphatic circulation as did that of the seventeenth and paid as much attention to it clinically, and physiologically, with the greater accuracy of theoretic scientific knowledge now possible, we might, within a very few years, attain practical results of the utmost importance.

The renaissance of the profession is attested in many ways, by a comparison of medical meetings twenty years ago and today. Broadly speaking, the profession has again become "the faculty" of a century ago. It is unified, strengthened, influential, able to control itself, to exercise a strong corrective and restraining power over individuals or cliques who placed their own selfish ends above the interests of the profession. It is no longer a suppliant for legislative favor, even in matters of the general welfare, but the acknowledged advisor of government in all matters in which medical experience and technical knowledge are required for sound judgment. Many of the evils and "problems" which were real, oppressive and apparently beyond our power to correct, even twenty years ago, have actually been settled, if not always perfectly, still to a reasonably satisfactory degree. Best of all, is the self-confidence gained, the conviction that there is no reform, present or future, necessary to the welfare of the profession or of the population at large and depending upon medical experience, which cannot be achieved by united effort.

Great advances are often indicated by apparently trivial details. Twenty years ago, consultation between a "Regular" and a Homoeopath, was an event. Forty years ago, two prominent members of these bodies, met at a social function. One remarked to the other, "Sir, we meet on neutral ground tonight"—and he spoke in all seriousness. Now, they would meet at a local society session as a matter of course, call each other by their first names, and talk over the latest case they had seen together, while waiting to discuss the same paper.

Fairly young men in the profession have witnessed the struggle of amalgamating small societies, limited almost to personal acquaintances, into strong local, scientific organizations; even younger ones have taken part in the welding together of the national, state and county societies and have watched the transition of united efforts at mutual protection and benefit from the stage at which it was denounced as chimeric, to actual successful operation. It is only a few years since the profession lent its aid, individually, to all sorts of impostures because the average physician had no information regarding the composition of a food or drug mixture, beyond the statements of the manufacturer. The writer can well remember being called visionary, because he suggested that the A. M. A. or some other body ought to analyse such preparations and render such information generally accessible. Today, we not only have information upon such matters, upon which the individual practitioner can base a judgment, but a fairly perfect legal control of the sale of drugs and foods and a practical elimination of the more serious offenders.

To return to the subject of medical meetings, how many of our recent graduates understand what is meant by a "Stormy Petrel?" Yet, twenty years ago, it was common medical slang to designate prominent doctors who never attended a meeting of a county or similar society unless there was a fight on—and they had a pretty good attendance record at that. Can they imagine a society of physicians that almost never pretended to hear a medical paper? Or that left, almost in a body, when the "business" session was over and the scientific session began, when such a session was planned at all? Could our young men master the intricate etiquette of seating themselves at meetings so as plainly to designate their political affiliations? Do they realize the recentness of a social session and lunch at the end of certain society meetings and that the generally attended dinners of others have grown out of the gatherings of a select few, at which the management of affairs was planned? Merely in attendance, our society meetings have shown in a comparatively few years, not only the development of a wholesome fraternal spirit, but a genuine interest in medicine rather than in medical politics. The man that we do not know is instinctively regarded as an enemy. Witness the history of ancient peoples and the attitude of a gang of little boys toward the new boy—each of us passes through the evolution of the race, on a small scale, in his own life. The profession seems to have passed out of the stage of barbarism and to be entering on a period of enlightenment when petty things will no longer distract its attention and when all of its energies will be concentrated on scientific and humanitarian problems.

